# Supplementary figures and images for: Systematic evaluation and validation of reference and library selection methods for deconvolution of cord blood DNA methylation data
Source: Clin Epigenetics. 2019 Aug 27;11:125. doi: 10.1186/s13148-019-0717-y (PMC6712867; doi:10.1186/s13148-019-0717-y)

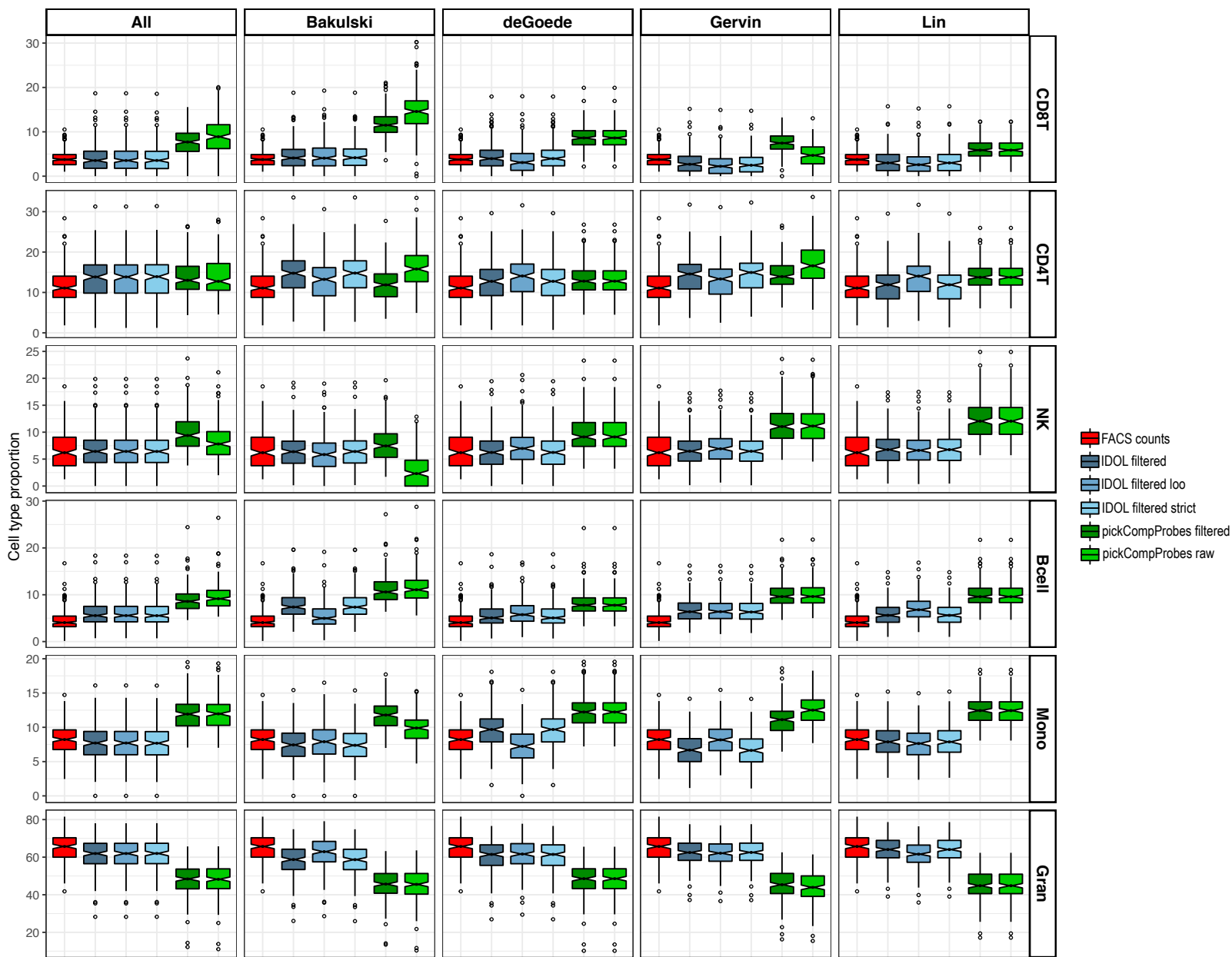

Supplement: Supplementary file 1 — Figure S1. Box plots of estimates and FACS counts. Cell type proportions generated by FACS (true values) and CP/QP programming (estimates) using IDOL and pickCompProbes L-DMRs for each UCB reference individually and combined. (PDF 110 kb) [file 13148_2019_717_MOESM1_ESM.pdf]

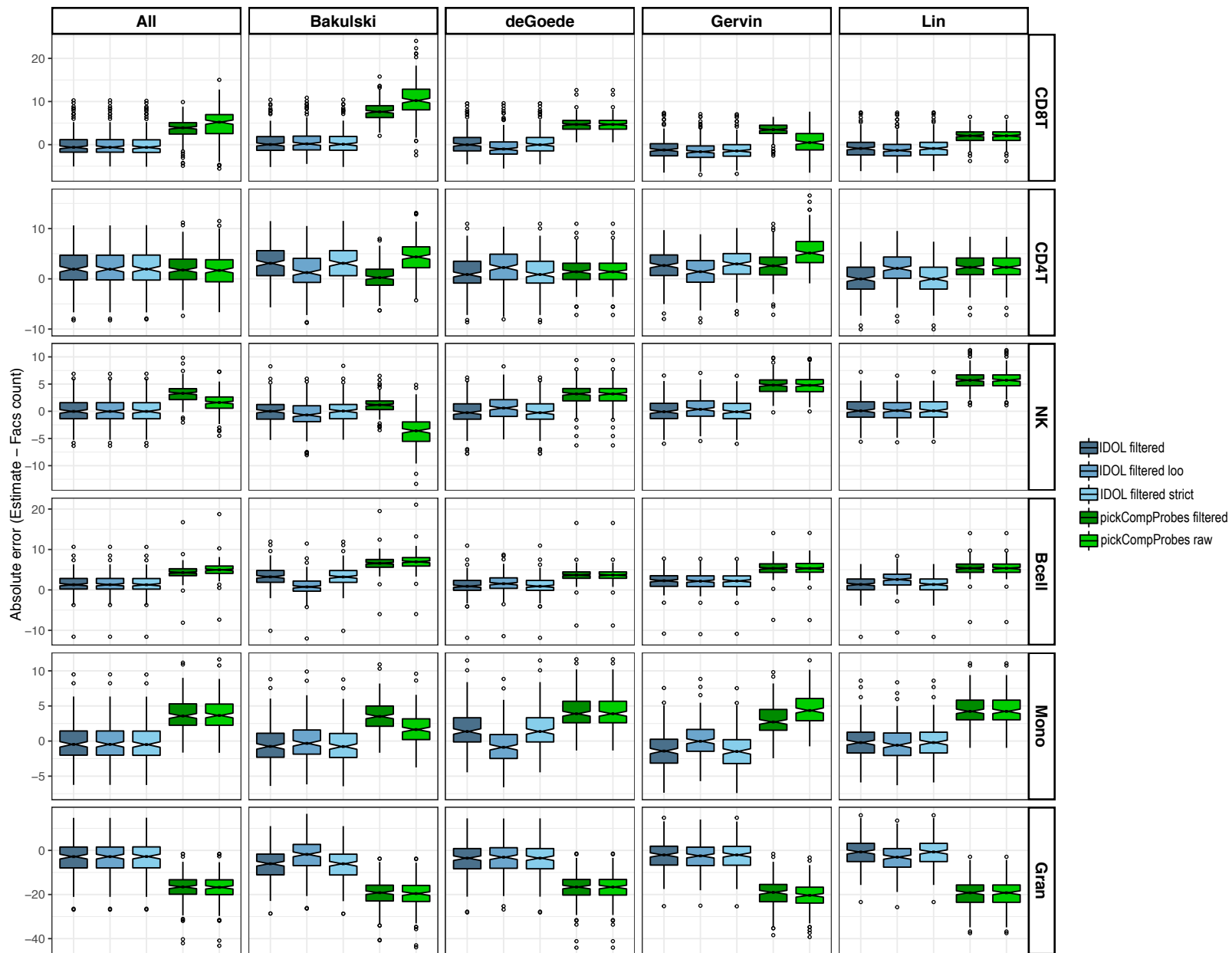

Supplement: Supplementary file 2 — Figure S2. Box plots of absolute errors. Absolute errors (estimates minus FACS counts) per method (IDOL and pickCompProbes) for each UCB reference individually and combined. (PDF 108 kb) [file 13148_2019_717_MOESM2_ESM.pdf]
